# Wind-mediated horseweed (*Conyza canadensis*) gene flow: pollen emission, dispersion, and deposition

**DOI:** 10.1002/ece3.1540

**Published:** 2015-06-17

**Authors:** Haiyan Huang, Rongjian Ye, Meilan Qi, Xiangzhen Li, David R Miller, Charles Neal Stewart, David W DuBois, Junming Wang

**Affiliations:** 1 Illinois State Water Survey, Prairie Research Institute, University of Illinois at Urbana-ChampaignChampaign, Illinois; 2 Department of Plant Sciences, University of Tennessee2431 Joe Johnson Dr., Knoxville, Tennessee; 3 School of Science, Wuhan University of TechnologyWuhan, Hubei, China; 4 Chengdu Institute of BiologyChengdu, Sichuan, China; 5 Department of Natural Resources and Environment, University of ConnecticutStorrs, Connecticut; 6 Department of Plant and Environmental Sciences, New Mexico State UniversityLas Cruces, New Mexico

**Keywords:** Atmosphere, deposition, dispersion, emission, horseweed, pollen, source strength

## Abstract

Horseweed (*Conyza canadensis*) is a problem weed in crop production because of its evolved resistance to glyphosate and other herbicides. Although horseweed is mainly self-pollinating, glyphosate-resistant (GR) horseweed can pollinate glyphosate-susceptible (GS) horseweed. To the best of our knowledge, however, there are no available data on horseweed pollen production, dispersion, and deposition relative to gene flow and the evolution of resistance. To help fill this knowledge gap, a 43-day field study was performed in Champaign, Illinois, USA in 2013 to characterize horseweed atmospheric pollen emission, dispersion, and deposition. Pollen concentration and deposition, coupled with atmospheric data, were measured in a source field (180 m by 46 m) and its surrounding areas up to 1 km downwind horizontally and up to 100 m vertically. The source strength (emission rate) ranged from 0 to 140 pollen grains per plant per second (1170 to 2.1×10^6^ per plant per day). For the life of the study, the estimated number of pollen grains generated from this source field was 10.5×10^10^ (2.3×10^6^ per plant). The release of horseweed pollen was not strongly correlated to meteorological data and may be mainly determined by horseweed physiology. Horseweed pollen reached heights of 80 to100 m, making long-distance transport possible. Normalized (by source data) pollen deposition with distance followed a negative-power exponential curve. Normalized pollen deposition was 2.5% even at 480 m downwind from the source edge. Correlation analysis showed that close to or inside the source field at lower heights (≤3 m) vertical transport was related to vertical wind speed, while horizontal pollen transport was related to horizontal wind speed. High relative humidity prevented pollen transport at greater heights (3–100 m) and longer distances (0–1000 m) from the source. This study can contribute to the understanding of how herbicide-resistance weeds or invasive plants affect ecology through wind-mediated pollination and invasion.

## Introduction

Glyphosate-resistant (GR) evolved weedy species biotypes (16 dicots and 15 monocots) have been identified worldwide (Heap 2014). Among which, horseweed (*Conyza canadensis*; a member of the Asteraceae family) has the most widespread distribution. Horseweed (*Conyza canadensis*) is considered to be a problem agricultural weed that can reduce soybean yield by 90% at high densities (Bruce and Kells 1990; Weaver 2001; Gibson et al. 2006; Davis and Johnson 2008). This species is native to North America and was the first eudicot weed to evolve GR, which was first detected in Delaware, USA, in 2000 (Van Gessel 2001). GR horseweed biotypes are now found on four continents and 24 US states (Heap 2014). Horseweed is commonly found in field and noncrop settings, where tillage has been reduced or eliminated. It often grows in association with other winter annuals (Shields et al. 2006). Horseweed grows in late spring, blooms, and produces seeds in August, September, and October, respectively. Plants can grow to a height of ∼0.8 m to ∼2.3 m, and a single plant can produce more than 200,000 seeds that are windborne with the aid of a pappus (Bhowmik and Bekech 1993; Weaver 2001). Horseweed seed is lightweight, with a gravitational-settlement velocity of 0.323 m/sec (Andersen 1993; Dauer et al. 2006). The spread of glyphosate-resistant biotypes has been rapid, with resistant populations covering greater than 44,000-ha in corn (*Zea mays* L.), soybeans, and cotton (*Gossypium hirsutum* L.) fields (Shields et al. 2006).

An increased understanding of the pollen and seed dispersion process would benefit the selection of strategies to control the spread of horseweed, especially resistant biotypes, across agricultural fields. Horseweed gene flow studies have been largely focused on seed spread (Dauer et al. 2006, 2007, 2009; Shields et al. 2006) instead of pollen transport, pollination, and outcrossing. The seeds can be lifted above 68–120 m altitude (Dauer et al. 2009). The seeds will potentially be carried for hours before descending (Dauer et al. 2009) and therefore would be transferred long distances. Horseweed is mainly self-fertilized; glyphosate-resistant (GR) horseweed can pollinate glyphosate-susceptible (GS) horseweed (4% outcrossing rate reported by Smisek 1995 and Henry et al. 2008). Gene flow via the transfer of GR via pollen, in addition to long-distance seed movement, is troubling because it could aid in the evolution of multiple resistance (Henry et al. 2008). However, there is little information available about its pollen production, atmospheric dispersion, and deposition.

Assessing and predicting pollen emissions can be challenging because the mechanisms of pollen release are sensitive to factors relative to plant biology, meteorological conditions, and local terrain (Menut et al. 2014). Usually, the pollen longevity is about 2 h (von Hout et al. 2008). Pollen production during flowering can vary by orders of magnitude from day-to-day, and the total volume of pollen grains released in a season can vary significantly year-to-year (Subiza et al. 1992; Emberlin et al. 1993). Pollen travel proximate to its source has been studied for many plant species to address pollen allergies (Holmes and Bassett 1963; Stark et al. 1997; Aboulaich et al. 2013) or gene flow in agricultural field crops (Llewellyn and Fitt 1996; Jarosz et al. 2003, 2005; von Hout et al. 2008). But studies of horseweed pollen emission and dispersion (either close to or far from the source) are lacking.

The objectives of this study were to: (1) measure atmospheric dynamic (on the order of an hour) horseweed pollen emission, dispersion, and deposition in the vertical direction (up to 100 m) and in the horizontal direction (up to 1000 m); and (2) quantify the correlation between horseweed pollen emission, dispersion, and deposition and atmospheric parameters.

## Materials and Methods

### Experimental site

**Figure 1:**
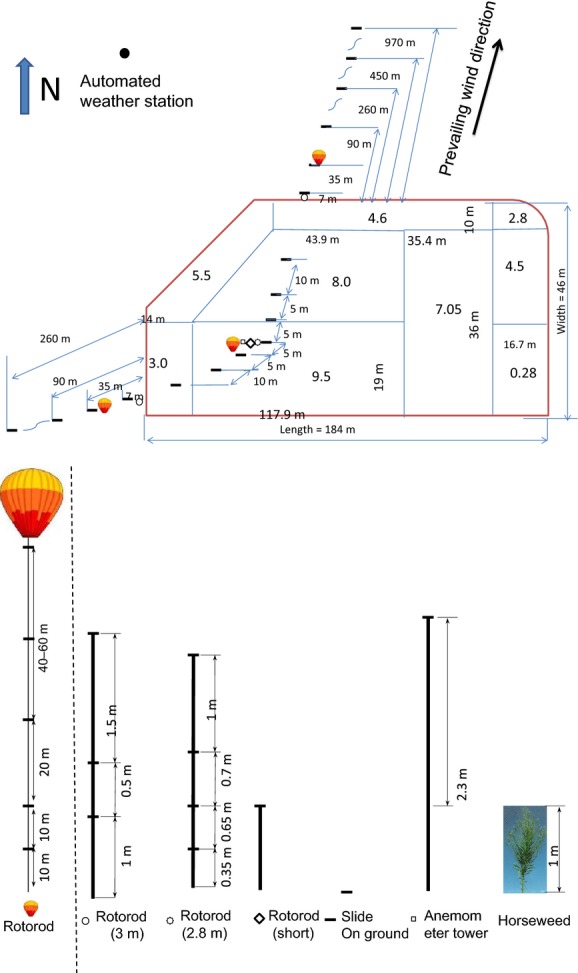
Schematic map and setup of the experiment. Number in each plot is plant density (plants/m^2^). In each experimental period, only the samplers (slide and Rotorod) along the downwind sampling line (northeast or southwest direction) were used. Balloon horizontal location and sampler heights on the balloons were adjusted during experiments based on if the pollen was detectable at the corresponding sampling heights and location.

The study was conducted from August 23, 2013 to October 12, 2013 on the South Research Farm, University of Illinois at Urbana-Champaign, Champaign, Illinois, USA (Latitude: 40° 04′ 51.36″ N; Longitude: 88° 14′ 23.92″ W; Elevation: 216 m). The experimental design consisted of a 184 m × 46 m plot of naturally occurring horseweed, hereafter called the source field (Fig.1). The field was surrounded by various grasses and soybeans. Within the source field, the average canopy height of horseweed was 1 m. On August 23, almost all of the horseweed plants (>95%) were fully mature and flowering. Therefore, when we calculated the source strength (emission rate), a percentage of the plants in flower were not accounted for. The flowering plants were evenly distributed spatially. There were no other horseweed plants within a 1000-m radius.

### Pollen concentration measurements

Pollen concentration was measured using columns of Rotorod samplers along the downwind direction (Fig.1). The prevailing wind direction was from southwest to northeast.

**Figure 2:**
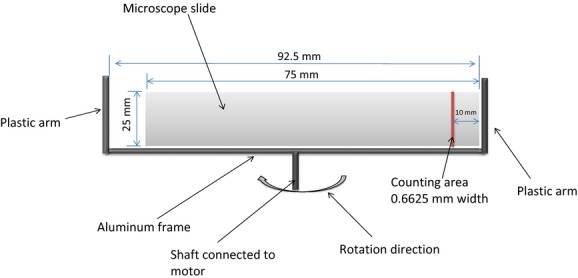
Schematic sketch of the sampling head of the Rotorod sampler.

One-column Rotorod samplers were placed in the source field to measure the horizontal flux (grains/m^2^/sec) profiles of source production and release, with one sampler placed inside the plant canopy at a height of 0.35 m, one at the height of the canopy (1 m), one at 1.65 m, and one at 2.8 m (Fig.1). On each Rotorod, pollen grains were collected on a transparent plastic microscope slide (width = 25 mm, length = 75 mm) that was fixed on a rotating rod (diameter = 92.5 mm) (Fig.2). The rod was attached to an electric motor. In order to retain the pollen grains, silicone grease was applied to the slide prior to sampling. The slide collection efficiency was assumed to be 64% and independent of wind speeds (Ogden and Raynor 1967). The microscope slides were changed to a fresh set every 2 to 3 h between 08:00 and 19:00. One additional sampler at the canopy height was placed in the source field to continuously record reference values of the source strength when the other samplers were switched off for rod replacement.

One column of three Rotorod samplers mounted at 1.0, 1.5, and 3.0 m was placed at the edge of the source field to measure downwind concentrations. The concentrations at higher heights (>3 m) were measured with the Rotorod samplers mounted below two balloons. The two balloons were in the downwind direction inside or outside of the field. The downwind distance of the balloons and the sampler heights were adjusted based on whether the pollen was detectable. The sampler heights ranged from 10 m to 100 m, and the balloon downwind distance ranged from inside the source field to 110 m downwind from the source.

### Pollen deposition measurements

The deposition rate of pollen in the source field and outside of the source field in the downwind direction was measured by greased microscope slides on the ground (the size of the slides was the same as those used in the Rotorod samplers). The deposition slides in the downwind direction were placed and collected at the same time as the concentration slides. The collection efficiency of the slide traps was assumed to be 100% (Raynor et al. 1972; Aylor and Ferrendino 1989; and Wang and Yang 2010). The Rotorod sampler slides and the deposition slides were not overloaded by the sampled pollen during each sampling period.

During rainy days, plants did not release pollen and experiments were not conducted.

### Meteorological measurements

3-D wind velocities were measured in the source field with a sonic anemometer placed at 1.3 m above the canopy (CSAT3; Campbell Sci, Logan, UT). The measurements were recorded at 10 Hz using a CR3000 data logger (Campbell Sci). Solar radiation, air temperature, relative humidity, and rainfall were measured by a weather station located about 800 m north of the source field every hour by the Water and Atmospheric Resources Monitoring Program at the Illinois State Water Survey, University of Illinois at Urbana-Champaign.

Parameters denoting atmospheric conditions were calculated from the weather station and the high-frequency anemometers for each sampling period after rotating the horizontal wind components into the mean wind direction (Wesely 1970). The parameters included friction velocity (u*, m/sec), atmospheric stability at anemometer height (3.3 m) (*ξ*(3.3)*,* unitless), mean vertical wind speed at anemometer height (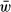 (3.3), m/sec) and its turbulent variability at 3.3 m (*σ*_*w*_(3.3), m/sec, i.e., standard deviation of vertical wind velocity), mean wind speed at 3.3 m 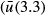, m/sec) and its standard deviation (*σ*_*u*_(3.3), m/sec), and wind direction at 3.3 m (*θ* (3.3), degree). A joint probability distribution of *θ* (3.3) and 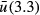 graphed as a wind rose was generated for each sampling period from the 3-D sonic data and used to project wind speed on the direction of the sampling lines. The atmospheric parameters of Monin–Obukhov length (L, m) and stability *ξ*(3.3) were calculated according to Stull (2001).

The instruments and heights used for the measurements of the meteorological variables are listed in Table1, which presents the averages and standard deviations of each meteorological variable during the whole study period.

**Table 1:** Statistics of meteorological variables collected in the experiment

Parameter	Symbol	Unit	Height (m)	Source	Mean ± standard deviation
Mean wind speed	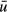 (3.3)	m/sec	3.3	Sonic anemometer	1.84 ± 0.69
Wind direction	Θ(3.3)	Degree	3.3	Sonic anemometer	228 ± 71
Mean vertical wind speed	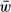 (3.3)	m/sec	3.3	Sonic anemometer	−0.03 ± 0.05
Friction velocity	u^*^	m/sec	3.3	Sonic anemometer	0.36 ± 0.12
Stability	*ξ*(3.3)	Unitless	*z* = 3.3	Sonic anemometer	−2.03 ± 3.75
Air temperature	*T*	°C	2.0	Weather station	25.42 ± 4.88
Relative humidity	RH	%	2.0	Weather station	54.21 ± 14.70
Solar radiation	SR	kw/m^2^	2.0	Weather station	0.43 ± 0.21
Rainfall	Rainfall	mm/h	2.0	Weather station	0.21 ± 2.00

### Data processing of concentration and deposition

Pollen grains on each slide were counted using a microscope. Following Wang et al. (2006), 8 random circular subareas (with a diameter (d) of 0.6625 mm each area, which is a view-field diameter of the microscope) on each deposition slide were counted, assuming the pollen was uniformly distributed on the slide. Horseweed pollen grains are prolate spheroid-shaped and have a spiked, rough surface. The polar axis (P) of horseweed pollen has an average axis length of 22 *μ*m, and the equatorial diameter (E) of the pollen grains has an average size of 16 *μ*m. The horseweed pollen shape and size were quite different from other pollen types. Therefore, it was very easy to distinguish them from other pollen.

Therefore, the pollen deposition rate (*D*) (grains/m^2^/sec) was determined as follows: 

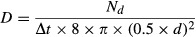
1*N*_d_ was the total number of pollen grains of the eight circular areas, 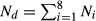, *N*_i_ was the number of grains in each of the circular areas, and *Δt* (sec) was the duration of each experimental period.

For the Rotorod sampling slides, the sampled pollen density decreased toward the rod center because the sampled air volume decreased with the decreased sampling radius. A significant portion of the pollen grains was deposited near the outside edge of the slides as noted earlier. A line of 0.6625 mm wide and 25 mm long at the edge was counted (Fig.2) following Wang and Yang (2009). The pollen concentration (C, grains/m^3^) was estimated as follows:

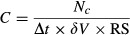
2

where *N*_c_ was the number of pollen grains within the selected line, RS was the rotation speed of Rotorod (revolutions/sec), and *δV* was the air volume sampled by the thin line during each revolution.

### Data processing of horizontal flux and source strength

**Figure 3:**
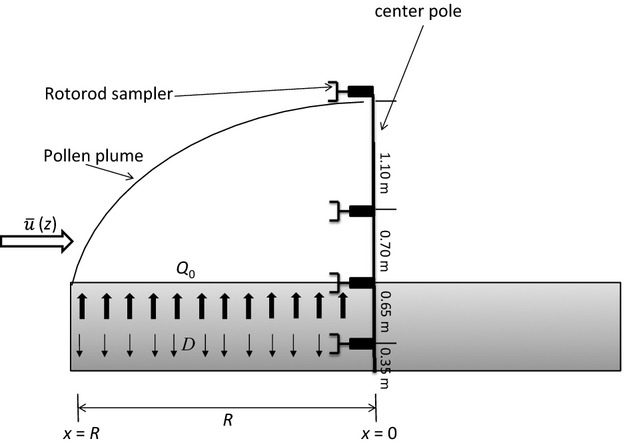
Schematic sketch of source strength measurement in a horseweed field, where *x* is the distance from the field Rotorod sampling column, R is the length of the field edge to the sampling column, 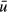 (*z*) is the horizontal wind speed, Q_0_ stands for pollen source strength, and D represents the downward deposition of pollen grains.

The horizontal flux of pollen at height *z* (*F*(*z*), grains/m^2^/sec) during each sampling period was calculated from pollen concentration at height *z*, *C*(*z*) (grains/m^3^), and wind speed at height *z* (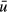 (*z*), m/sec), as *F*(*z*) = *C*(*z*) 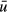 (*z*) (Fig.3). Wind speeds at different heights were calculated from the formulations in Campbell and Norman (1998), which are based on the atmospheric similarity theory (inputs were u*, *ξ*(*z*), and height *z*). The integrated horizontal flux (IHF) was estimated by integrating *F*(*z*) using the trapezoidal method. The integrand was zero at the ground because the concentration and wind speed at *z* = 0 m were zero.

The pollen source strength is a measure of the amount of pollen produced per unit area or plant per unit time, which is a determinant of the pollen dispersal distance. Following Griffith et al. (2008) and Wang and Yang (2009), source strength Q_0_ (grains/plant/sec) was calculated as (Fig.3):

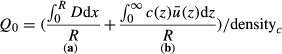
3where term a was the contribution of deposition, term b was the contribution of IHF, R was the distance between the leading edge of the field to the location of concentration sensors in the wind direction, and density_c_ was the plant density at the source center (10 plants/m^2^). This equation assumes that the source area is of uniform properties. However, as shown in Fig.1, the plant density of the field varied from about 10 plants/m^2^ at the center of the field to less than 2 plants/m^2^ at the edge of the source field. In order to take into account the effect of the variation of plant density, we divided the *R* into several segments Δ*R*_i_ at different density areas, and then scaled the Δ*R*_*i*_ to Δ*R*_scaled, *i*_ with respect to the corresponding plant density: 

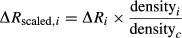
4where density_c_ is the density at the center of the field (9.5 plants/m^2^) as mentioned before and density_*i*_ is the density at the location *i*. The *R* in eq. 3 is the summation of Δ*R*_scaled, *i*._

### Data analysis

Correlation analyses were conducted to examine the effects of atmospheric parameters on pollen dispersal parameters. Atmospheric parameters included u*, *ξ*(3.3), 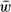 (3.3), *σ*_*w*_(3.3), 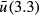, and *σ*_*u*_(3.3) in the sampling directions, air temperature (*T*) and its standard deviation (*σ*_T_), solar radiation (SR), and relative humidity (RH). Pollen dispersal parameters included pollen concentration (C) and deposition (D) in the center of the field, IHF and source strength, *Q*_*o*_ (representing source production), the ratio of center concentration at different heights to at the canopy height (pollen vertical transport), the ratio of concentration at the field edge to that at the field center canopy height (horizontal transport), the ratio of deposition at different distances to that at the field’s center (horizontal transport), the ratio of balloon-measured concentration at different heights to the center concentration at canopy height (vertical transport), and the ratio of the balloon-measured concentration at different downwind distances to the center concentration at canopy height (horizontal transport). All data analyses were conducted with the commercial software Minitab release 13 (Minitab, 2000).

## Results

### Concentration and deposition in the source plot

**Figure 4:**
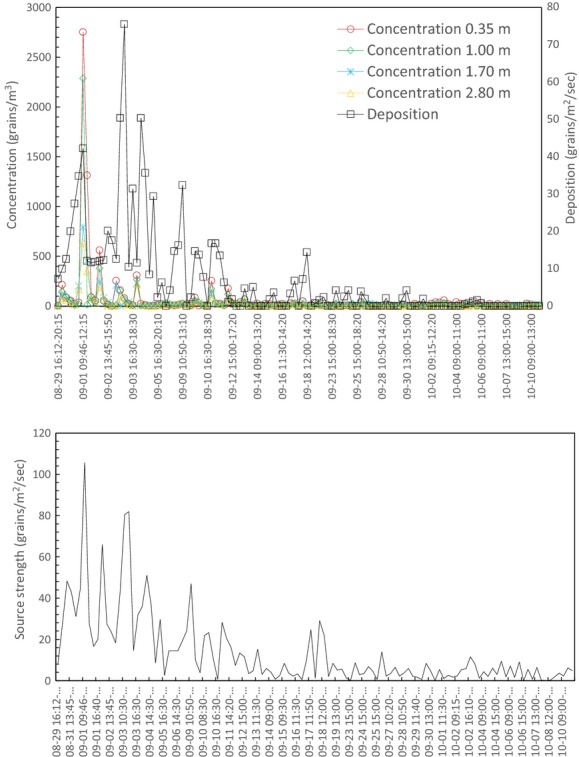
Pollen concentration (at 0.35 m, 1 m, 1.7 m and 2.8 m heights) and deposition at the source center (location of the column of the Rotorod samplers), and pollen source strength during each experimental period.

During the study, 121 concentration and deposition samples were collected in the source field. As shown in Fig.4, the concentration of pollen grains ranged from 0 to 2750 grains/m^3^ and deposition ranged from 0 to 76 grains/m^2^/sec.

**Figure 5:**
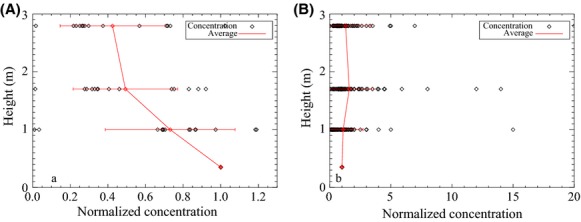
Vertical distribution of concentration at the source plot, (A) concentration at 0.35 m ≥ 50 grains/m^3^, (B) concentration at 0.35 m < 50 grains/m^3^.

Figure5(A) shows a typical profile of normalized concentration from a surface source (C ≥ 50 grains/m^3^), which decreases exponentially with height (data were normalized by the concentration at 0.35 m height). In Fig.5(B) (C < 50 grains/m^3^), the profile shows no significant change with height. This may be due to the samplers’ lack of precision at very low concentrations, or the pollen in the air was not actively released from the canopy, and ambient pollen was just being moved around randomly so the whole layer became well mixed.

### Concentration and deposition outside of the source field

**Figure 6:**
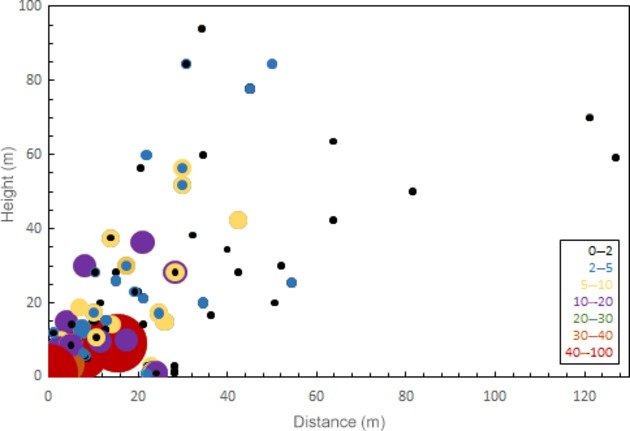
Pollen concentration (grains/m^3^) vs downwind distance and height from the location of the column of Rotorod samplers in the field. The points at distance = 0 m at different height are the averages of the data measured at the corresponding height at distance = 0 m during the whole season (showing the averages because the data were too crowded); other data are the data measured during each experimental period. The field edge in the southwest sampling line was at 22 m from the location and the edge in the northeast sampling line was 30 m.

As distance/height from the source increased, pollen concentration gradually decreased. As shown in Fig.6, pollen grains were found at heights of 80–100 m (0–8 grains/m^3^), which was about 0–12.5% of the concentration at the source (63–100 grains/m^3^). Therefore, pollen grains can be dispersed at a high altitude. At or beyond the field edge, many concentrations in the air were on the order of 0–3 grains/m^3^ which was approximately 0–5% of the concentration at the source. However, occasionally, concentrations on the order of 3–8 (2.8–12.5%) or 8–16 grains/m^3^ (7–25%) were found downwind out to 120–130 m from the source field (Fig.6).

**Figure 7:**
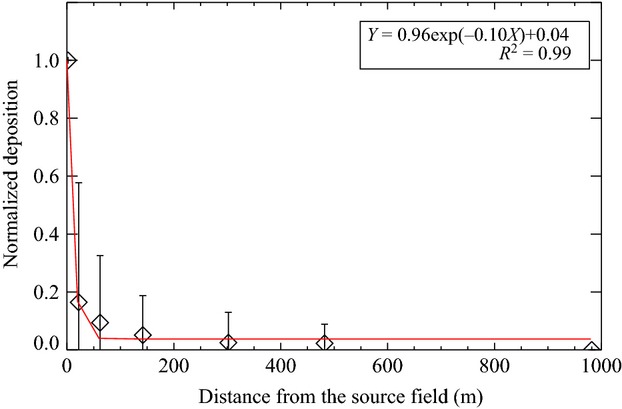
Pollen deposition along the downwind direction, *x*-axis is distance from the source field edge. Pollen deposition was normalized by the source deposition average at the source field center.

Pollen deposition with distance followed a negative-power exponential curve (Fig.7). The deposition decreased to 17% at 7 m from the source field edge. Then, deposition gradually decreased with distance. Even at 300 to 480 m, the average deposition was 2.5% compared to that at the source. At 1000 m, pollen deposition decreased to 0.

### Source strength

The source strength in the study was in the range of 0–140 pollen grains per plant per second (Fig.4). The peak of diurnal variation occurred at 11:00 to 13:00.

The number of pollen grains emitted per day ranged from 1170 to 2.1 × 10^6^ per plant. Over the study period, the average pollen production was 2.3 × 10^6^ grains per plant, which corresponds to about 10.5 × 10^10^ grains for the whole source plot.

### Influence of meteorological factors

#### Source production

Pearson correlation coefficients (*r*) of pollen parameters in the field and meteorological parameters are presented in Table2. Bonferroni corrections were used to judge if the coefficients were statistically significant, that is, because there were 10 correlation coefficients for each pollen variable (e.g., *C*_1_). Nonsignificance was defined as *P* > 0.005 (common used *P* > 0.05 was revised to *P* > 0.05/10). Source strength was not significantly correlated to meteorological parameters. This means that the release of horseweed pollen may be mainly determined by horseweed physiology instead of by atmospheric effects, that is, the plants themselves control when to release pollen and the amount of pollen to release. This may also explain the fact that the concentration (C_1_ to C_4_), deposition, and IHF in the source were weakly (¦*r*¦ ≤ 0.5, *P* < 0.005) or were not at all significantly correlated to atmospheric parameters (*P* > 0.005).

**Table 2:** Correlation coefficient (*r*) of meteorological and pollen parameters at the source field. ‘C_*i*_’ (*i* = 1 to 4) is the concentration in the source plot (grains/m^3^), C_1_ is at 2.8 m, C_2_ 1.7 m, C_3_ 1.0 m, and C_4_ 0.35 m; ‘CE_i_’ (i = 1to 3) is the concentration at the edge of the source field, CE1 is at 3.0 m, CE_2_ 1.5 m, and CE_3_ 1.0 m. Deposition (grains/m^2^/sec) is data collected in the center of the field at 0.35 m height. IHF is the integrated horizontal flux (grains/m^2^/sec) at the center of the field. u^*^: friction velocity, m/sec; *ξ*(3.3): atmospheric stability at anemometer height (3.3 m), unitless; 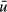
(3.3): mean wind speed at anemometer height (3.3 m), m/sec; 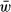 (3.3): mean vertical wind speed at anemometer height (3.3 m), m/sec; *T*: air temperature; RH: relative humidity, %;SR: solar radiation; ‘*σ*’ means standard deviation of the corresponding meteorological parameter

Variable	Number of sample	u^*^	*ξ*(3.3)	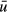 (3.3)	*σ*_*u*_ (3.3)	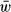 (3.3)	*σ*_*w*_ (3.3)	*T*	*σ*_*T*_	RH	SR
C_1_	121	−0.16 (NS)	−0.19 (NS)	−0.17 (NS)	−0.18 (NS)	0.04 (NS)	0.01 (NS)	0.06 (NS)	−0.13 (NS)	0.35 (***)	0.00 (NS)
C_2_	121	−0.17 (NS)	−0.18 (NS)	−0.19 (NS)	−0.19 (NS)	0.01 (NS)	0.07 (NS)	0.00 (NS)	0.07 (NS)	0.38 (***)	0.07 (NS)
C_3_	121	−0.12 (NS)	−0.11 (NS)	−0.13 (NS)	−0.17 (NS)	0.07 (NS)	−0.02 (NS)	−0.05 (NS)	−0.15 (NS)	0.23 (NS)	0.01 (NS)
C_4_	121	−0.13 (NS)	−0.16 (NS)	−0.16 (NS)	−0.19 (NS)	0.07 (NS)	0.00 (NS)	0.03 (NS)	−0.17 (NS)	0.31 (***)	−0.03 (NS)
Deposition	121	−0.16 (NS)	−0.06 (NS)	−0.20 (NS)	0.00 (NS)	−0.50 (***)	−0.01 (NS)	0.20 (NS)	−0.08 (NS)	0.06 (NS)	0.17 (NS)
IHF	121	−0.08 (.38)	−0.21 (.02)	−0.11 (.22)	−0.18 (NS)	0.01 (NS)	0.02 (NS)	0.02 (NS)	0.08 (NS)	0.36 (***)	0.02 (NS)
Source strength	121	−0.12 (NS)	−0.09 (NS)	−0.19 (NS)	−0.05 (NS)	−0.60 (NS)	–0.00 (NS)	0.16 (NS)	–0.09 (NS)	0.22 (NS)	0.245 (NS)
C_1_/C_3_	121	0.30 (NS)	0.05 (NS)	0.35 (***)	0.20 (NS)	−0.53 (***)	0.26 (NS)	0.04 (NS)	0.20 (NS)	0.07 (NS)	−0.21 (NS)
C_2_/C_3_	121	0.25 (NS)	0.08 (NS)	0.30 (NS)	0.11 (NS)	−0.56 (NS)	0.24 (NS)	−0.01 (NS)	0.25 (NS)	−0.04 (NS)	−0.19 (NS)
C_4_/C_3_	121	0.17 (NS)	0.02 (NS)	0.19 (NS)	0.13 (NS)	−0.37 (***)	0.16 (NS)	0.21 (NS)	0.45 (***)	0.11 (NS)	−0.25 (NS)
CE_3_/C_3_	17	0.73 (***)	0.11 (NS)	0.74 (***)	0.34 (NS)	−0.64 (NS)	0.27 (NS)	−0.34 (NS)	0.05 (NS)	−0.18 (NS)	0.03 (NS)
CE_2_/C_3_	17	0.73 (***)	0.11 (NS)	0.74 (***)	0.34 (NS)	−0.64 (NS)	0.27 (NS)	−0.18 (NS)	0.05 (NS)	−0.18 (NS)	0.02 (NS)
CE_1_/C_3_	17	0.83 (***)	0.15 (NS)	0.84 (***)	0.50 (NS)	−0.76 (***)	0.35 (NS)	−0.22 (NS)	0.20 (NS)	−0.12 (NS)	−0.12 (NS)

NS, not significant: *P* > 0.005

*** Significance level: *P* < 0.0001.

#### Pollen vertical transport

Pollen vertical transport (C_1_/C_3_, C_2_/C_3_, and C_4_/C_3_) in the source field at a low height (≤3 m) was weakly positive (*r* < 0.4, *P* < 0.005) or not significantly related to wind speed (*P* > 0.005), u* or standard deviation of air temperature, while negatively and moderately (¦*r*¦ > 0.5 and <0.7) or weakly (¦*r*¦ < 0.4) related to vertical wind speed. This indicates that the main atmospheric parameter affecting vertical transport of pollen in the source field is vertical wind speed. This was also shown in the strong (¦*r*¦ > 0.7) correlation of vertical wind speed and the ratio of above canopy concentration at the field edge to the canopy level at the field center (CE_1_/C_3_). The stronger the vertical speed, the more pollen was transported in the vertical direction, the smaller the ratio of the vertical concentration.

#### Pollen horizontal transport

From the source field to the source edge, the pollen horizontal transport (CE_3_/C_3_, CE_2_/C_3_, and CE_1_/C_3_) was positively and strongly (*r* > 0.7) related to the horizontal wind speed and u*, while it (CE_1_/C_3_) was strongly and negatively related to vertical wind speed (Table2). As expected, this implies that stronger horizontal wind can bring more source pollen to the field edge, while stronger vertical wind mixed more pollen in the vertical direction and reduced the proportion of the pollen amount with height.

The horizontal concentration ratio outside of the field was partially and negatively related to relative humidity (Table3, *r* = −0.95 at 40–60 m downwind). This implies that humidity in the air may have prevented horizontal pollen transport.

**Table 3:** Correlation coefficient (p value) of meteorological parameter and the ratio (concentration at different downwind distance to canopy concentration at field center). (30 m<downwind concentration measurement height ≤100 m). u^*^: friction velocity, m/sec; *ξ*(3.3): atmospheric stability at anemometer height (3.3 m), unitless; 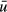 (3.3): mean wind speed at anemometer height (3.3 m), m/sec; 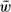 (3.3): mean vertical wind speed at anemometer height (3.3 m), m/sec; *T*: air temperature; RH: relative humidity, %; SR: solar radiation; ‘*σ*’ means standard deviation of the corresponding meteorological parameter

Horizontal distance	Number of sample	u^*^	*ξ*(3.3)	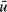 (3.3)	*σ*_u_ (3.3)	*T*	*σ*_*T*_	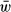 (3.3)	*σ*_w_ (3.3)	RH	SR
0–20 m	4	0.69 (NS)	0.87 (NS)	0.59 (NS)	−0.06 (NS)	0.14 (NS)	−0.10 (NS)	0.36 (NS)	−0.35 (NS)	0.83 (NS)	−0.68 (NS)
20–40 m	10	0.08 (NS)	0.48 (NS)	−0.02 (NS)	−0.19 (NS)	0.25 (NS)	0.06 (NS)	0.18 (NS)	−0.18 (NS)	0.27 (NS)	−0.50 (NS)
40–60 m	7	−0.24 (NS)	−0.66 (NS)	−0.27 (NS)	−0.33 (NS)	−0.33 (NS)	−0.15 (NS)	0.19 (NS)	0.09 (NS)	−0.95 (***)	0.27 (NS)
60–140	4	−0.10 (NS)	0.65 (NS)	−0.28 (NS)	−0.90 (NS)	−0.59 (NS)	−0.56 (NS)	0.89 (NS)	−0.76 (NS)	0.78 (NS)	−0.89 (NS)

NS, not significant: *P* > 0.005

*** Significance level: *P* < 0.0001.

## Discussions

Information on gene flow through pollen dispersion for self-pollinating plants is lacking because researchers believe that the outcrossing ratio is low. Although the gene flow through pollination is low compared with seed dispersion, gene flow from herbicide-resistant weeds through pollination has the potential to produce multiple herbicide-resistant weeds (Heap 2014). Even for cross-pollinating plants (e.g., genetically modified plants), there is not much information on pollen dynamic release, dispersion, and deposition. The amount of information on long-distance and high altitude dispersion is even less, and studies on the relationship of dynamic pollen release and dispersion with atmospheric factors are few. Therefore, it is important to obtain such gene flow information. This paper reported such information for horseweed. The methodology related to horseweed in this study can be adapted for other weeds or invasive plants. The information in the study can aid in the understanding of the invasion and competition of weeds and other invasive plants in ecological systems. Therefore, it will be helpful for managing weeds or invasive plants.

### Concentration and source strength in the source field

The range of horseweed pollen concentration at canopy height in our study was similar to the experimental data for timothy in 1966, as reported in Raynor et al. (1972) (2,750 grains/m^3^ vs. 0–2542 in Table4). However, the range was 2.5 to 15 times smaller than in the 1962 and 1963 timothy data and 1.7 times smaller than in the ragweed data (Raynor et al. 1972) (Table4). Compared to the corn pollen experiments in Wang and Yang (2010) and Raynor et al. (1972), the range of horseweed pollen concentration was much larger (Table4).

**Table 4:** Comparison of the literature pollen release data to this study. Settling speed was calculated based on Stokes’ Law, 2014 and pollen density in von Hout et al. 2008

Plants	Source dimension	Plant density	Pollen diameter	Pollen settling speed	Concentration at canopy at source center	Source strength
	m	Plants/m^2^	*μ*m	m/sec	Grains/m^3^	Grains/plant/sec
Horseweed (This study)	184 by 46 m	9.5	16–22	0.017	0–2750	0–140
Timothy Raynor et al. (1972)	36.3 m diameter in 1962	Planted densely, no density data	30–35	0.037	0–11,000	Not available
	36.3 m diameter in 1963	Planted densely, no density data	30–35	0.037	0–50,500	Not available
	18.3 m diameter in 1966	Planted densely, no density data	30–35	0.037	0–2542	Not available
Ragweed Raynor et al. (1972)	Point source	Not available	18–22	0.018	0–5400	Not available
Corn Wang and Yang (2010)	16 m diameter	7	90–100	0.3	0–1200	0–73
Corn Raynor et al. (1972)	18.3 diameter in 1963	7.5	90–100	0.3	0–100	Not available
	18.3 diameter in 1964	3	90–100	0.3	0–54	Not available

The horseweed pollen release rate (0–140 grains/plant/sec) was greater than corn plant’s 0–73 pollen grains/plant/sec during a pollination season (Wang and Yang 2010). The peak of diurnal pollen release (source strength) occurred around noon (11:00–13:00). The pattern was similar to the typical pattern found in wind-pollinated plants, with the maximum pollen source release occurring at late morning, 10:00–10:30 (Scott 1970; Jarosz et al. 2003; Wang and Yang 2009). At this time of day, relative humidity was low, solar radiation was not strong enough to kill pollen, and the atmosphere was turbulent, all of which resulted in a relatively long, viable dispersion time with wide dispersion by the turbulent air.

The differences of pollen concentrations and source strengths between this study and those in the literature may be due to environmental factors (atmospheric condition, topography), plant types, and measurement methods. Limited experiments cannot provide complete sensitivity distributions of pollen emissions to all these possible forcing variables. But these results will provide useful parameterizations for modeling studies of pollen release.

### Long-distance and high altitude transport

Pollen dispersal may occur over varying distances; for instance, sugar beet pollen can be found more than 1000 m from the source, while most corn pollen travels a distance no more than 100 m (Eastham and Sweet 2001; Bots and Mariani 2005). The pollen dispersal distance and pattern depend on the amount of pollen produced by plants, release height, meteorological conditions, and settling velocity.

In this study, concentration and deposition generally decreased with elevation and/or distance, but due to the light weight of horseweed pollen, the decreased trends were not as sharp as in the previous studies of corn pollen (Jarosz et al. 2005; von Hout et al. 2008). For example, Jarosz et al. (2005) found that at the distance of 100 m, corn pollen deposition decreased to 1–10% (pollen diameter: 90–100 *μ*m, settling speed 0.3 m/sec). Yet, in this study the deposition was still 2.5% at 480 m downwind (because pollen diameter is 16–22 *μ*m, (Ye et al. 2014) and settling speed 0.0165 m/sec were smaller than corn’s, Table4).

With a slightly larger pollen diameter (30–35 *μ*m, settling speed 0.037 m/sec), timothy pollen dispersal was similar to horseweed’s. Timothy had 24.4% deposition at 36.6 m compared to the source deposition, while horseweed had 18% deposition at 25 m.

### Influence of meteorological factors

#### Pollen vertical transport and pollen horizontal transport

Similar to our study, the importance of wind in determining the dispersal distance was noted by Jarosz et al. (2005) and Raynor et al. (1972). The pollen travel distance increased with higher wind speed.

High relative humidity may have prevented pollen transport (negative correlation in Table3). This is consistent with findings in the literature (Stennett and Beggs 2004; Aboulaich et al. 2013). Aboulaich et al. (2013) stated that pollen can absorb moisture in the air, which makes pollen heavier and easier to settle.

#### Potential outcrossed seeds at different distances

Horseweed is mainly self-fertilized. During the pollination season, first the flowers were closed and pollination occurred inside its female stigmata. After most of the ovules were fertilized, the flowers opened and released the pollen. After about 10 to 12 days, the seeds were mature. Smisek (1995) and Henry et al. (2008) found that adjacent GR and GS horseweeds had an outcrossing ratio of 4%. If the pollen outcrossing ratio was linearly related to pollen deposition like corn (Wang et al. 2006), based on the deposition measured in the study, outcrossed seeds with a 0.1% outcrossing ratio (=4 × 2.5% deposition at 260 to 480 m) may be found at 260–480 m from the source if GS stands were there. The horseweed produced 31,140 seeds/plant (Huang et al. 2014). Then at 260 to 480 m, the outcrossed GS plants may produce 31 outcrossed seeds/plant (=31,140 × 0.1%). This amount may cause serious problems with the spread of GR plants. The risk should be determined with future experiments.

#### Source strength

The favorable meteorological conditions that can promote pollen release include low humidity, high temperature, unstable atmosphere, strong wind, and little precipitation. The effects of meteorological factors on the release of pollen may differ, depending on the local climatic features and topography, as well as the type of plant (Norris-Hill and Emberlin 1991; Vázquez et al. 2003; Aboulaich et al. 2013). In this study, horseweed pollen production was not significantly related to atmospheric parameters and was controlled mainly by its physiology.

## Data accessibility

This section will be added at the paper revision stage. The data will be archived at: http://datadryad.org/.

## References

[b1] Aboulaich N, Achmakh L, Bouziane H, Trigo MM, Recio M, Kadiri M (2013). Effect of meteorological parameters on Poaceae pollen in the atmosphere of Tetouan (NW Morocco). Int. J. Biometeorol.

[b2] Andersen MC (1993). Diaspore morphology and seed dispersal in several wind-dispersed Asteraceae. Am. J. Bot.

[b3] Aylor DE, Ferrendino FJ (1989). Dispersion of spores released from an elevated line source within a wheat canopy. Boundary Layer Meteorol.

[b4] Bhowmik PC, Bekech MM (1993). Horseweed (*Conyza canadensis*) seed production, emergence, and distribution in no-tillage and conventional tillage corn (*Zea mays*. Agron. Trends Agric. Sci.

[b5] Bots M, Mariani C (2005). Pollen viability in the field.

[b6] Bruce JA, Kells JJ (1990). Horseweed (*Conyza canadensis*) control in no-tillage soybeans (Glycine max) with preplant and preemergence herbicides. Weed Technol.

[b7] Campbell GS, Norman JM (1998). An introduction to environmental biophysics: 2nd edition.

[b8] Dauer JT, Mortensen DA, Humston R (2006). Controlled experiments to predict horseweed (*Conyza canadensis*) dispersal distances. Weed Sci.

[b9] Dauer JT, Mortensen DA, VanGessel MJ (2007). Temporal and spatial dynamics of long-distance *Conyza canadensis* seed dispersal. J. Ecol.

[b10] Dauer JT, Mortensen DA, Luschei EC, Isard SA, Shields E, VanGessel MJ (2009). *Conyza canadensis* seed ascent in the lower atmosphere. Agric. For. Meteorol.

[b11] Davis VM, Johnson WG (2008). Glyphosate-resistant horseweed (*Conyza canadensis*) emergence, survival, and fecundity in no-till soybean. Weed Sci.

[b12] Eastham K, Sweet J (2001). http://www.e-library.lt/resursai/ES/Leidiniai/EEA_issue_reports/GMOsforwww.pdf.

[b14] Emberlin J, Savage M, Woodman R (1993). Annual variations in the concentrations of Betula pollen in the London area, 1961–1990. Grana.

[b15] Gibson KD, Johnson WG, Hillger DE (2006). Farmer perceptions of weed problems in corn and soybean rotation systems. Weed Technol.

[b16] Griffith DW, Bryant GR, Hsu D, Reisinger AR (2008). Methane emissions from free-ranging cattle: comparison of tracer and integrated horizontal flux techniques. J. Environ. Qual.

[b17] Heap IM (2014). http://www.weedscience.org.

[b18] Henry RS, Davis VM, Johnson WG (2008). http://www.btny.purdue.edu/weedscience/Postslide/Henry08-01.pdf.

[b19] Holmes R, Bassett I (1963). Effect of meteorological events on ragweed pollen count. Int. J. Biometeorol.

[b20] von Hout R, Chamecki M, Brush G, Katz J, Parlange M (2008). The influence of local meteorological conditions on the circadian rhythm of corn (*Zea mays* L.) pollen emission. Agric. For. Meteorol.

[b21] Huang H, Ye R, Peng Y, Wang J, Stewart CN (2014). Seed and pollen dispersion and invasion from Round-Up-resistant horseweed (Conyza canadensis).

[b22] Jarosz N, Loubet B, Durand B, McCartney A, Foueillassar X, Huber L (2003). Field measurements of airborne concentration and deposition rate of maize pollen. Agric. For. Meteorol.

[b23] Jarosz N, Loubet B, Durand B, Foueillassar X, Huber L (2005). Variations in maize pollen emission and deposition in relation to microclimate. Environ. Sci. Technol.

[b24] Llewellyn D, Fitt G (1996). Pollen dispersal from two field trials of transgenic cotton in the Namoi Valley, Australia. Mol. Breeding.

[b25] Menut L, Vautard R, Colette A, Khvorostyanov D, Potier A, Hamaoui-Laguel L (2014). A new model of ragweed pollen release based on the analysis of meteorological conditions. Atmos. Chem. Phys. Discuss.

[b500] MINITAB (2000).

[b26] Norris-Hill J, Emberlin J (1991). Diurnal variation of pollen concentration in the air of north-central London. Grana.

[b27] Ogden EC, Raynor GS (1967). New sampler for airborne pollen the rotoslide. J. Allergy.

[b28] Raynor G, Ogden E, Hayes J (1972). Dispersion and Deposition of Corn Pollen from Experimental Sources. Agron. J.

[b29] Rayor GS, Hayes JV, Ogden EC (1970). Experimental data on dispersion and deposition of timothy and corn pollen from known sources.

[b30] Scott RK (1970). The effect of weather on the concentration of pollen within sugar-beet seed crops. Ann. Appl. Biol.

[b31] Shields EJ, Dauer JT, Van Gessel MJ, Neumann G (2006). Horseweed (*Conyza canadensis*) seed collected in the planetary boundary layer. Weed Sci.

[b32] Smisek AJJ (1995).

[b33] Stark P, Ryan L, McDonald J, Burge H (1997). Using meteorologic data to predict daily ragweed pollen levels. Aerobiologia.

[b34] Stennett PJ, Beggs PJ (2004). Pollen in the atmosphere of Sydney, Australia, and relationships with meteorological parameters. Grana.

[b35] Stokes’ Law (2014). http://en.wikipedia.org/wiki/Stokes%27_law.

[b36] Stull RB (2001). An introduction to boundary kayer meteorology.

[b37] Subiza J, Masiello JM, Subiza JL, Jerez M, Hinojosa M, Subiza E (1992). Prediction of annual variations in atmospheric concentrations of grass pollen. A method based on meteorological factors and grain crop estimates. Clin. Exp. Allergy.

[b38] Van Gessel MJ (2001). Glyphosate-resistant horseweed from Delaware. Weed Sci.

[b39] Vázquez LM, Galán C, Domínguez-Vilches E (2003). Influence of meteorological parameters on olea pollen concentrations in Crdoba (South-western Spain). Int. J. Biometeorol.

[b40] Wang J, Yang X (2009). Improved method for nondestructive measurement of dynamic pollen source strength from transgenic crops using sonic anemometer. Int. J. Agric. Biol. Eng.

[b41] Wang J, Yang X (2010). Development and validation of atmospheric gene flow model for assessing environmental risks from transgenic corn crops. Int. J. Agric. Biol. Eng.

[b42] Wang J, Yang X, Li Y, Elliot PF (2006). Pollination competition effects on gene-flow estimation: Using regular vs. male-sterile bait plants. Agron. J.

[b43] Weaver SE (2001). The biology of Canadian weeds.115. *Conyza canadensis*. Can. J. Plant Sci.

[b44] Wesely ML (1970).

[b45] Ye R, Peng Y, Wang J, Millwood RJ, Stewart CN (2014).

